# Fire, CO_2_, and climate effects on modeled vegetation and carbon dynamics in western Oregon and Washington

**DOI:** 10.1371/journal.pone.0210989

**Published:** 2019-01-25

**Authors:** Tim Sheehan, Dominique Bachelet, Ken Ferschweiler

**Affiliations:** 1 Conservation Biology Institute, Corvallis, Oregon, United States of America; 2 Graduate School, Oregon State University, Corvallis, Oregon, United States of America; 3 Department of Biological and Ecological Engineering, Oregon State University, Corvallis, Oregon, United States of America; Oak Ridge National Laboratory, UNITED STATES

## Abstract

To develop effective long-term strategies, natural resource managers need to account for the projected effects of climate change as well as the uncertainty inherent in those projections. Vegetation models are one important source of projected climate effects. We explore results and associated uncertainties from the MC2 Dynamic Global Vegetation Model for the Pacific Northwest west of the Cascade crest. We compare model results for vegetation cover and carbon dynamics over the period 1895–2100 assuming: 1) unlimited wildfire ignitions versus stochastic ignitions, 2) no fire, and 3) a moderate CO_2_ fertilization effect versus no CO_2_ fertilization effect. Carbon stocks decline in all scenarios, except without fire and with a moderate CO_2_ fertilization effect. The greatest carbon stock loss, approximately 23% of historical levels, occurs with unlimited ignitions and no CO_2_ fertilization effect. With stochastic ignitions and a CO_2_ fertilization effect, carbon stocks are more stable than with unlimited ignitions. For all scenarios, the dominant vegetation type shifts from pure conifer to mixed forest, indicating that vegetation cover change is driven solely by climate and that significant mortality and vegetation shifts are likely through the 21^st^ century regardless of fire regime changes.

## Introduction

Expected ecosystem responses to climate change include altered fire regimes (e.g. [1–3]), insect outbreaks (e.g. [4]), hydrologic changes (e.g. [5]), altered nutrient cycling (e.g. [6]), species range shifts (e.g. [7–9]), and novel species assemblages (e.g. [10–11]). Vegetation models have been used to simulate such changes and provide resource managers projections to help their decision process [12]. Estimating associated uncertainty allows managers to modulate their strategies [12].

Dynamic Global Vegetation Models (DGVMs) are process-based models that simulate vegetation, carbon, nutrient, and hydrological dynamics. They are driven by historical climate data and climate projections from General Circulation Models (GCMs; e.g. [13]) or Earth System Models (ESMs; [14]). Sources of uncertainty in DGVM projections come from both external drivers such as climate and soil characteristics, and internal characteristics such as model structure, empirical parameter values, built-in thresholds, and inherent assumptions and simplifications.

Another source of uncertainty is the complex relationship between fire and vegetation, which takes place over a range of spatial and temporal scales [15]. Shifts in fire regime cause vegetation-altering feedbacks (e.g. [16–17]). The type and level of complexity of fire models adequate for management-relevant vegetation modeling remains unclear [18]. Researchers have implemented a variety of models [19–21] which may or may not include fuel types, fuel moisture, ignitions sources, fire suppression, rate of spread, and energy release component calculation [18, 22]. Disturbance modeling at the landscape scale is discussed in [23], and fire model limitations and uncertainties in global vegetation models are described in [18, 22]. While comparing results among DGVMs using different fire models provides one way to characterize uncertainty, modifying the assumptions within a single DGVM’s fire model is another method for sensitivity analysis and the exploration of fire-related uncertainty.

An additional source of uncertainty is the assumptions of CO_2_ effects on plant productivity. CO_2_ concentration effects on the water use efficiency and productivity of many species is not well known (e.g. [24]). Increased productivity has been attributed to the CO_2_ fertilization effect, but plant responses at large scales, with complex species assemblages, and combined with concurrent warming are uncertain [25]. Free-air CO_2_ enrichment experimental results (FACE) [26] have shown that increased CO_2_ can cause an increase in water use efficiency (WUE), leaf area index (LAI) and net primary productivity (NPP), but other factors, such as nutrient availability, may constrain responses over time (e.g. [27]). Species-specific response can modulate plant responses (e.g. [27–28]) and increased NPP may not increase C stocks [24] just as increased WUE may not always lead to increased growth [29]. Uncertainties in the CO_2_ fertilization effect underscore the importance of testing different assumptions with DGVMs to explore vegetation response.

A previous study simulated climate change effects on fire and vegetation in the Pacific Northwest using the MC2 DGVM [1]. That study characterized the uncertainty due to different atmospheric CO_2_ concentrations, climate drivers, and anthropogenic fire suppression actions. Fire occurrence and effects were driven by fuel condition thresholds and unlimited ignition sources. That study used a modest CO_2_ fertilization effect proportional to atmospheric CO_2_ concentration.

In this study, we evaluate uncertainty due to model assumptions regarding fire occurrence and CO_2_-driven WUE. We compare results from unlimited ignitions and fixed fuel thresholds to those with stochastic ignition occurrence and ignition propagation based on fuel conditions. We also compare results obtained with and without CO_2_ fertilization effect. We address the following research questions concerning vegetation and carbon dynamics in the MC2 DGVM:

1. What are the consequences of model assumptions about wildfire ignitions on spatial and temporal fire effects, carbon dynamics, and vegetation dynamics?
2. What are the consequences of model assumptions about CO_2_ fertilization effects on carbon and vegetation dynamics?

## Methods

### Study area

**Fig 1:**
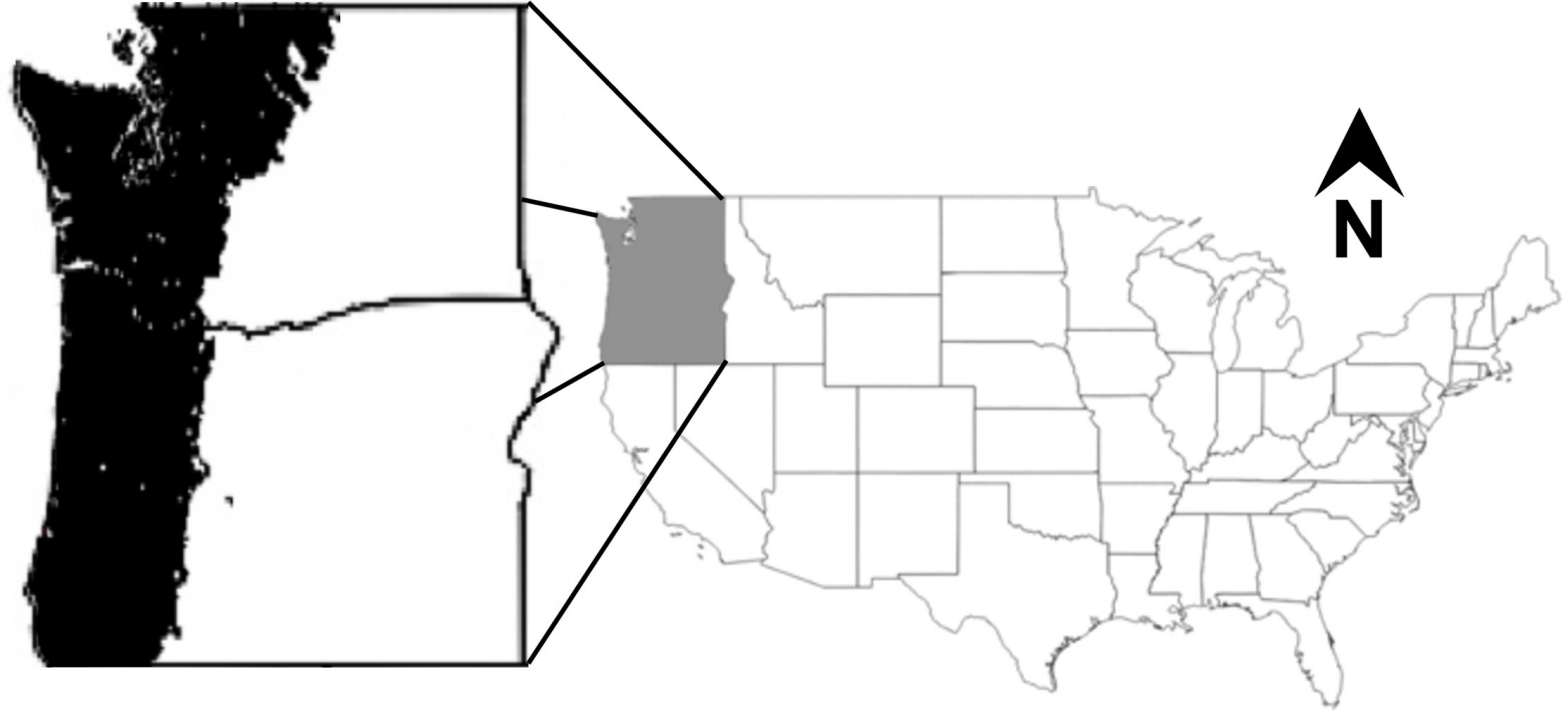
Study area. Portions of Oregon and Washington west of the Cascade Mountain Range crest.

The study area (Fig 1) consists of portions of Oregon and Washington west of the Cascade Mountain Range crest that include Coast Range, Klamath Mountains/California High North Coast Range, Willamette Valley, Puget Lowlands, Cascades, and North Cascades Level III Ecoregions [30]. This area falls under strong coastal influence with mild, wet winters and dry summers.

### Model description

We used the MC2 dynamic global vegetation model (DGVM) [31] to simulate potential vegetation shifts, carbon fluxes, and wildfires. We simulated potential vegetation without land use effects, and with previously defined model parameterization and protocol for the conterminous United States (detailed in [31]).

MC2 does not simulate species, but instead simulates combinations of life forms in functional vegetation types. Woody lifeforms (trees and shrubs) are distinguished by leaf phenology (evergreen vs. deciduous) and morphology (needleleaf vs. broadleaf). The woody lifeforms and the relative dominance of C3 versus C4 grasses (including sedges and forbs) are simulated using climate thresholds. Carbon thresholds are used to distinguish broad vegetation types ranging from forest to grassland.

The fire module simulates fire occurrence and fire effects including area burned, mortality, consumption of aboveground biomass, carbon emissions, and nitrogen volatilization. Fire occurrence is simulated as a discrete event. The module runs on a pseudo-daily time step and derives a randomly distributed set of daily precipitation amounts from monthly precipitation values. Fuel types are derived from carbon stocks, and their characteristics are determined by weather effects on their moisture content (see [1] for a detailed description). Per vegetation type fire return intervals (FRIs) and time since last fire are used to limit the maximum portion of a grid cell burned. Fire occurrence is based on fuel condition thresholds and assumed unlimited ignitions. Fire suppression is simulated by assuming fires below empirical fuel condition thresholds can be extinguished while those above cannot.

For this study, we added an optional, three-stage stochastic ignition algorithm to MC2. Stage one uses a per-day ignition source probability and a Monte Carlo draw to determine whether a grid cell is exposed to an ignition source. Stage two checks fuel conditions for fine fuels moisture code (FFMC) [32] and buildup index (BUI) [33]. With unlimited ignitions, both FFMC and BUI must exceed a cell’s vegetation type’s threshold for a fire to be simulated. With the stochastic algorithm, they must both exceed a specified fraction of their respective thresholds. The third stage uses a Monte Carlo method to determine whether or not an ignition source initiates a fire. The probability of fire initiation is determined using the Chapman-Richards function:

$$s={\left(1-e^{\left(-k*ffmc\_thresh\_frac\right)}\right)}^{2}$$ (1)

where *ffmc_thresh_frac* is defined as the fraction of the FFMC threshold, adjusted to offset the curve so that values below the minimum threshold produce an initiation probability of zero, and values near the maximum threshold produce a initiation probability near 1.0. It is calculated as:

ffmc_thresh_frac=max((ffmc_min_frac–ffmc_max_frac),0) (2)

*k* in (2) is the Chapman-Richards constant and is calculated as:

$$k=\frac{-ln(1-\sqrt{0.99})}{ffmc\_max\_frac–ffmc\_min\_frac}$$ (3)

where *ffmc_min_frac* is the fraction of the FFMC threshold below which fire initiation approaches 0, and *ffmc_max_frac* is the fraction of the FFMC threshold where it approaches 1.

**Fig 2:**
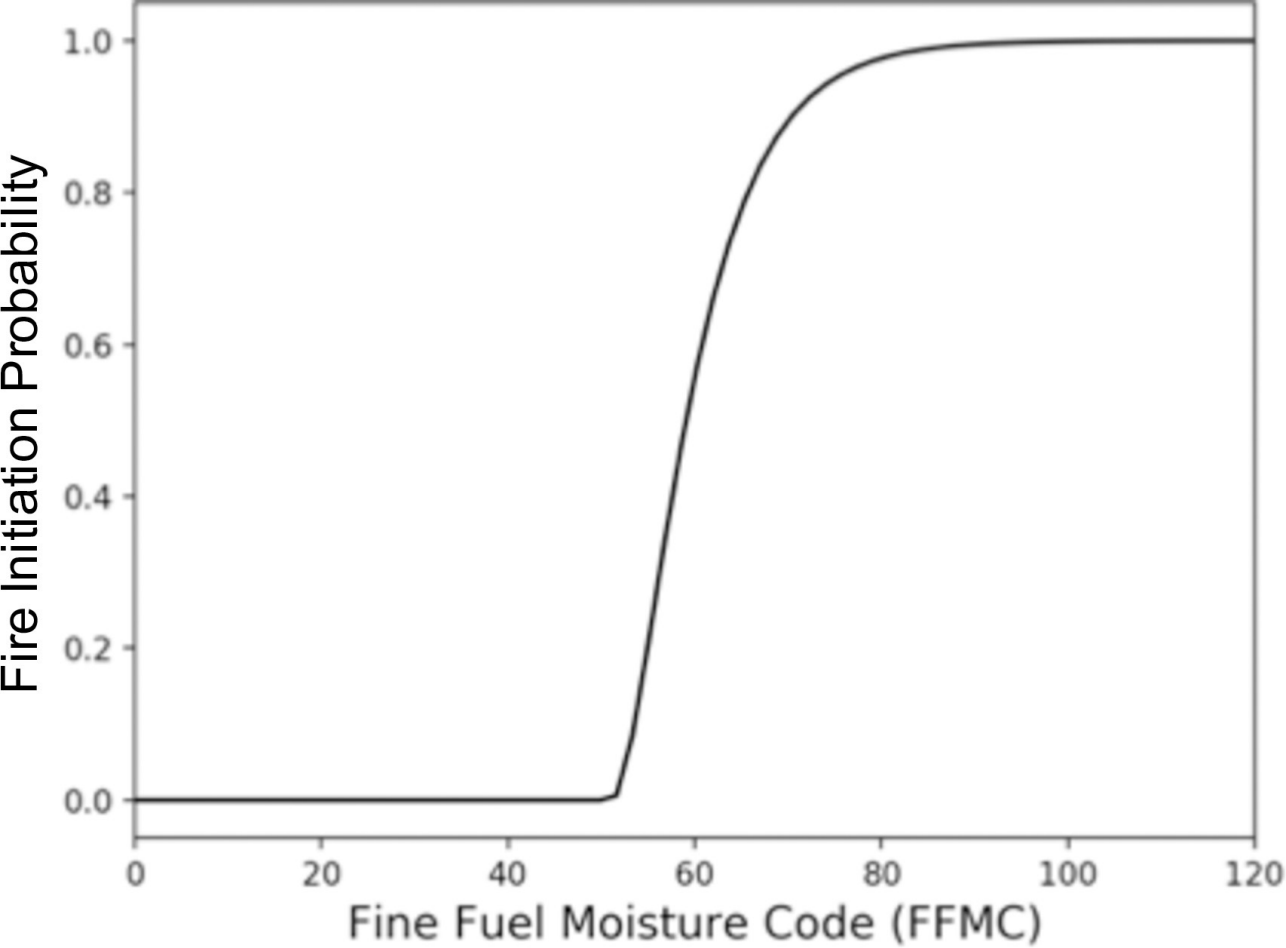
Example fire initiation probability curve. This example is for a vegetation type with a fine fuel moisture code (FFMC) threshold of 86, an FFMC and buildup index (BUI) minimum threshold fraction of 0.6 and an FFMC maximum threshold fraction of 1.0.

For this study, the daily ignition source probability was 0.001, the *threshold_fraction* was 0.6 and *ffmc_min_frac* and *ffmc_max_frac* were 0.6 and 0.99 respectively (Fig 2).

**Fig 3:**
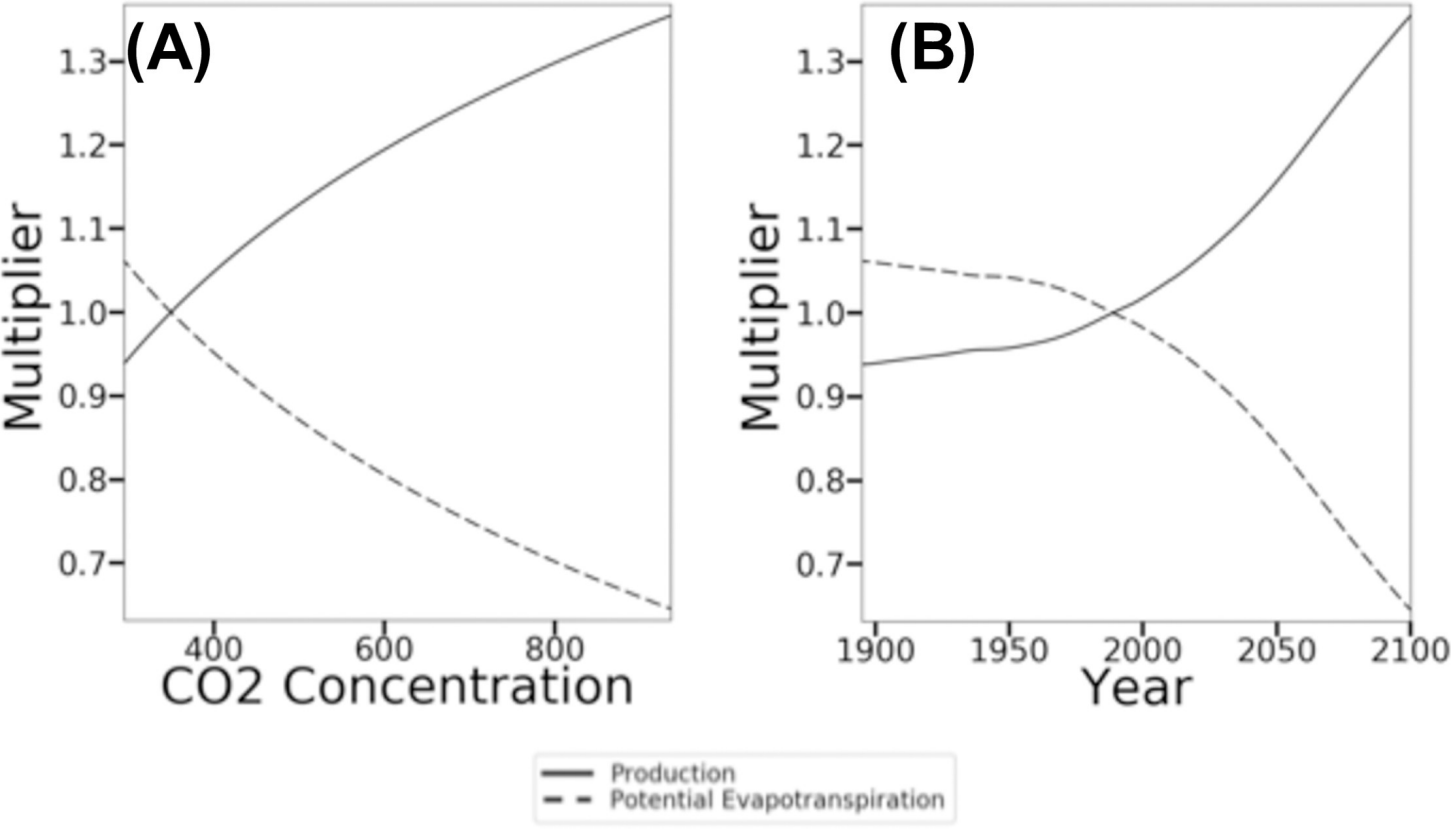
CO_2_ fertilization effect scalar. The scalar used in MC2 to calculate production and potential evapotranspiration vs (A) atmospheric CO_2_ concentration and (B) year.

To implement the CO_2_ fertilization effect on WUE, MC2 uses a multiplier applied directly to production and transpiration. It is calculated as:

$$multiplier=1+(effect_{param}-1)*{log}_{2}\frac{current\_co2\_conc}{baseline\_co2\_conc}$$ (4)

where *multiplier* is the value used to modify production, *effect_param* specifies the degree of the effect, *current_co2_conc* is the CO_2_ concentration for the current model year, and *baseline_co2_conc* is the CO_2_ concentration at which the multiplier is equal to 1.0 (350 ppm in this study). CO_2_ concentrations above 350 ppm yield a positive effect and values below 350 yield a negative effect. The default CO_2_ fertilization effect used in this study is 1.25 (Fig 3).

### Model runs

We ran MC2 on a 1/24 degree (~4 km) grid using PRISM [34] data for the historical period (1895–2010) and CCSM4 (National Center for Atmospheric Research) climate projections for 2011–2100 downscaled using the MACA [35] algorithm which performs well in capturing fire danger indices across the western US. We used the CO_2_ concentrations associated with RCP 8.5 (“business as usual”). We used the same CMIP5 climate, CO_2_ projections, and soil data as in [1].

### Run protocol

For this study, we ran the model with different combinations of fire and CO_2_ fertilization effects (Table 1). To run without the CO_2_ fertilization effect, atmospheric CO_2_ concentration was held at its preindustrial value.

**Table 1:** Fire and CO_2_ fertilization scenarios used for this study’s MC2 runs.

	With CO_2_ fertilization effect (WCE for with CO_2_ fertilization effect)	Without CO_2_ fertilization effect (NCE for no CO_2_ fertilization effect)
**Assumed ignitions, without fire suppression (FF for full fire)**	FF-WCE	FF-NCE
**Assumed ignitions with fire suppression (FS)**	FS-WCE	Not modeled
**Stochastic fire (SF)**	SF-WCE	Not modeled
**No fire (NF)**	NF-WCE	NF-NCE

### Validation and comparison with other studies

For the FF-WCE and SF-WCE scenarios, we compared simulated results with the observed area burned from the Monitoring Trends in Burn Severity (MTBS) [36] fire perimeter dataset (https://www.mtbs.gov/) dataset. We also compared simulated results for aboveground live woody biomass (AGB), aboveground dead woody carbon (AGD), and total aboveground woody carbon (AGT) densities and pools with published modeled results based on observed Forest Inventory and Analysis (FIA) National Program (https://www.fia.fs.fed.us/) data ([37], hereafter, Hudiburg, available on Data Basin, http://bit.ly/2CcZ7wK; [38,14,39]. To reflect the influence of land use, we limited our results to non human-affected (NHA) based on the LandFire US 140 EVT dataset (landfire.gov; 30m x 30m).

In the Hudiburg datasets, we set densities of carbon in human-affected (HA) cells to 0 before resampling to the 1/24 degree grid used in our simulation. We adjusted carbon densities from our simulation results by multiplying the results by the ratio of NHA area to the total grid cell area. We similarly adjusted results for validation against the MTBS [36] dataset.

### Analyses

We compared fire results, carbon dynamics, and vegetation change among modeled scenarios. For fire, we compared three results: 1) area with fire (AWF)–the total area of grid cells burned; 2) fraction area burned (FAB)–fraction of area burned in grid cells with fire; and 3) total area burned (TAB), the sum of (AWF * FAB) over all grid cells.

For carbon, we compared live and dead carbon (C) pools, total ecosystem C stocks, net primary production (NPP), net ecosystem production (NEP), net biome production (NBP), and C consumed and emitted by fire (consumed C). Results for C pools and fluxes were summarized by taking mean values over the study area for five 30-year periods: early 20^th^ c. (1895–1924), mid 20^th^ c. (1936–1965), late 20^th^ c. (1971–2000), mid 21^st^ c. (2036–2065), and late 21^st^ c. (2071–2100).

To more easily compare vegetation cover, we reclassified vegetation types into four categories: conifer forest; temperate mixed conifer/broadleaf forest; subtropical mixed conifer/broadleaf forest; and other which includes vegetation types dominated by grasses, forbs, and shrubs (S1 Table). We then calculated the mode of the vegetation category for each grid cell for each time period and calculated the area-weighted distribution for each category.

### Background climate description

Climate projections used for the future period (2011–2100) are overall hotter with decreasing summer precipitation towards the late 21^st^ c. and increasing PET. Maximum annual and April-September average temperatures are relatively constant over the 20^th^ c. (S1A Fig, S2 Table), with 30-year means varying by 0.2°C or less. During the 21^st^ c. temperatures increase sharply with maximum annual temperatures 4.4°C higher, and April-September average temperatures 4.8°C higher than during the late 20^th^ c. Annual precipitation increases by 4% from the early to late 20^th^ c. and an additional 6% from the late 20^th^ c. to the late 21^st^ c. (S1B Fig, S2 Table). April-September precipitation increases 6% over the 20^th^ c. but compared to the late 20^th^ c., decreases by 15% during the mid 21^st^ c. and again during the late 21^st^ c. (S1B Fig, S2 Table). PET (calculated by MC2) increases by 3% from the early to the late 20^th^ c., increases by 29% from the late 20^th^ c. to the mid 21^st^ c. and by 52% from the early 20^th^ c. to the late 21^st^ c. (S1C Fig, S2 Table).

## Results

### Validation and comparison with other studies

**Fig 4:**
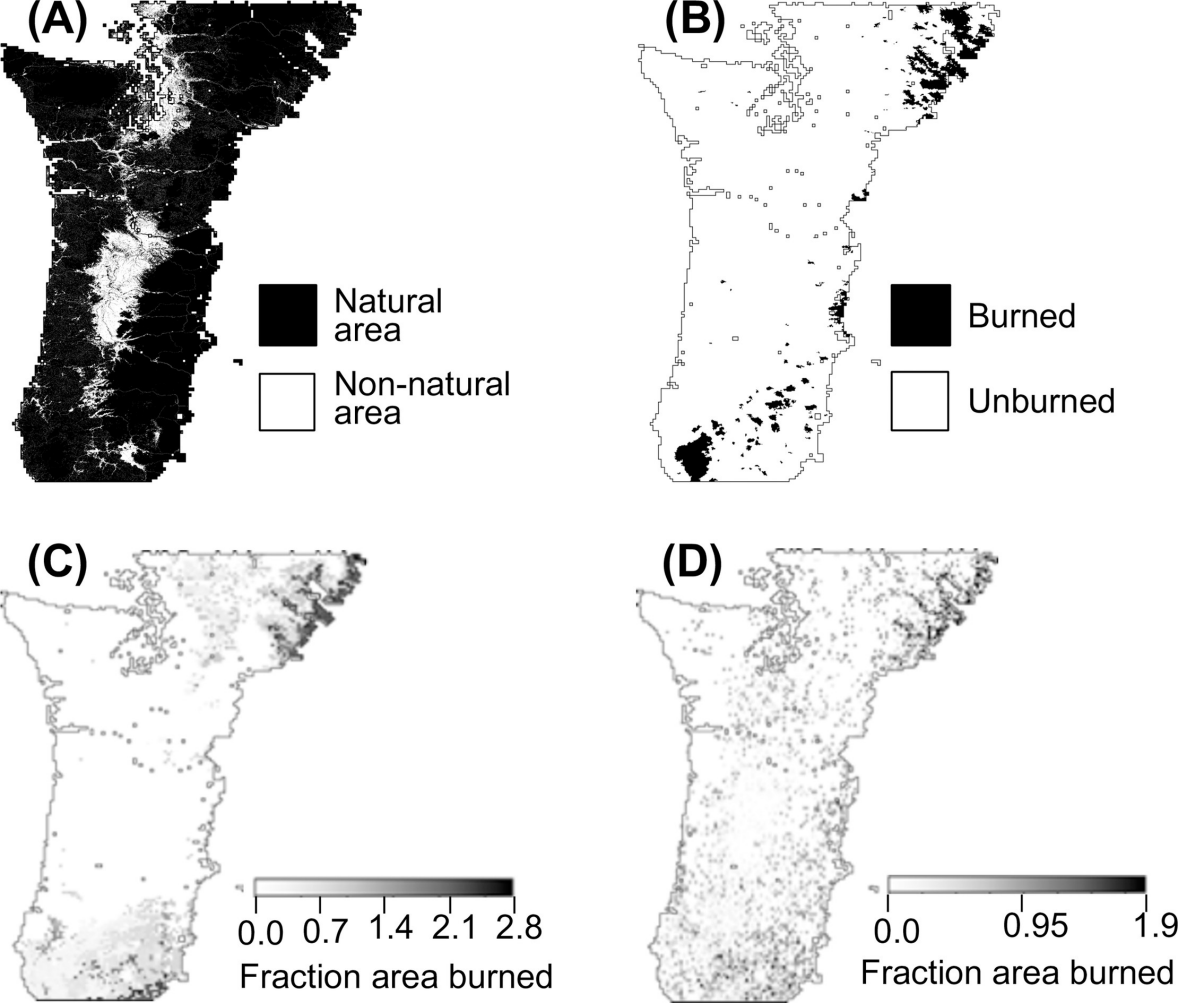
Measures of fire on the landscape. (A) Natural and human-affected areas as determined by reclassifying LandFire vegetation classes; (B) MTBS fire perimeters for 1985–2015; (C) Total NHA (non-human affected) area burned over 1985–2015 (sum of FAB weighted by grid cells’ NHA fraction over 1985–2015) for FF-WCE (full fire, with CO_2_ fertilization effect); and (D) Total NHA area burned over 1985–2015 for SF-WCE (no fire suppression, with CO_2_ fertilization effect).

For simulations with fire, TAB (total area burned) is 2.3 to 2.8 times observed (Table 2). Burned areas for both observed and FF-WCE are concentrated across the southeastern corner of the study area, the central east edge, and the northeastern corner (Fig 4B and 4C). However, for FF-WCE, fire is simulated in the northernmost central portion of the area (northern Cascades) where it is not observed, and in the southeast fire is less concentrated than observed. For SF-WCE, fire occurrence is also concentrated in the southeastern and northeast (Fig 4D), but also occurs more frequently throughout non-human-influenced areas than either for observed or FF-WCE, most commonly in the Cascade Mountains, southern Coast Range, and Puget Trough (Fig 4B–4D).

**Table 2:** Area burned over the period 1985–2012 for MTBS and TAB (total area burned, AWF (area with fire) * FAB (fraction burned)) for with-fire simulations.

**Scenario**	**Area Burned (km**^**2**^**)**
MTBS (km^2^)	10,753
FF-WCE (km^2^)	28,205
FS-WCE (km^2^)	24,828
SF-WCE (km^2^)	24,603
FF-NCE (km^2^)	29,211

(FF-WCE: full fire, with CO_2_ fertilization effect; FS-WCE: with fire suppression, with CO_2_ fertilization effect; SF-WCE: with stochastic ignitions, with CO_2_ fertilization effect; and FF-NCE: full fire, with no fertilization effect)

**Fig 5:**
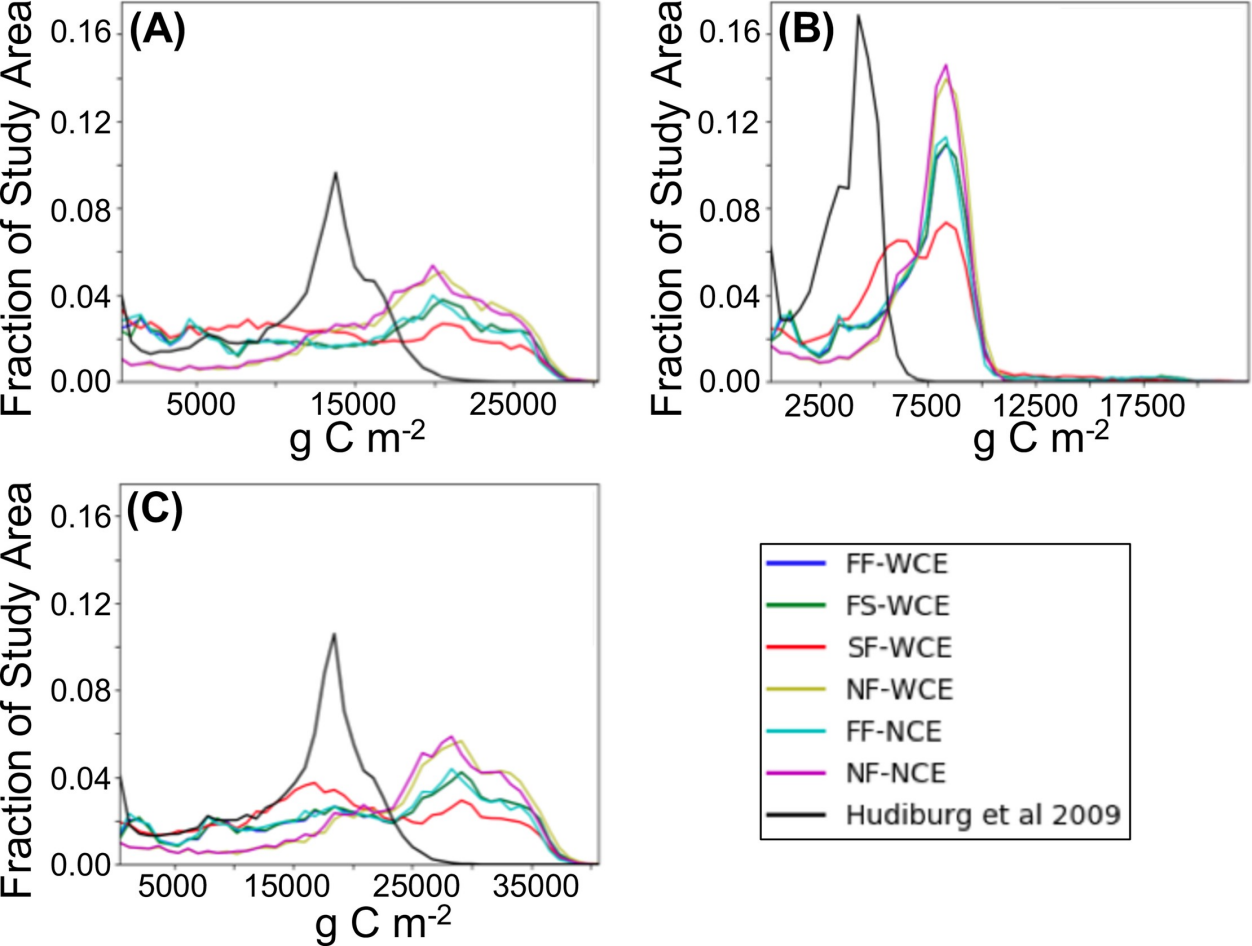
Simulated carbon density distributions by the MC2 vegetation model for natural areas as a fraction of the entire study area. (A) AGB (above ground live woody biomass), (B) AGD (above ground dead woody carbon), and (C) AGT (above ground total woody carbon).

Our simulated AGB ranges from 11 to 57% higher than that modeled by Hudiburg (Table 3). AGD carbon also ranges from 79 to 105% higher, and AGT carbon ranges from 28 to 68% higher. Among our simulations, AGB, AGD, and AGT are highest for NF-WCE, and lowest for SF-WCE (Table 3). AGB, AGD, and AGT densities are generally higher and more flatly distributed than Hudiburg’s (Fig 5). AGB, AGD, and AGT density distributions are flattest for SF-WCE.

**Table 3:** Carbon values for Hudiburg and MC2 results.

**Veg. Type**	**AGB Total (Pg)**	**AGD Total (Pg)**	**AGT Total (Pg)**	**AGB Densitiy** **(gCm**^**-2**^**) (maximum)**	**AGD Density** **(gCm**^**-2**^**)** **(maximum)**	**AGT Density** **(gCm**^**-2**^**)** **(maximum)**
**Hudiburg**	1.84	0.58	2.42	26900–44200 (~50000-~70000)	2600–9500 (~8000-~1700)	38100–46800 (~58000-~84000)
**FF-WCE**	2.31	1.11	3.43	14205	6826	21092
**FS-WCE**	2.32	1.12	3.44	14266	6887	21154
**SF-WCE**	2.05	1.04	3.09	12606	6395	19001
**NF-WCE**	2.89	1.19	4.07	17772	7318	25028
**FF-NCE**	2.26	1.09	3.35	13897	6703	20600
**NF-NCE**	2.82	1.17	3.99	17341	7195	24536

NHA (non-human affected) AGB (above ground live woody biomass), AGD (above ground dead woody carbon), and AGT (above ground total woody carbon). (For Hudiburg values, absolute values were calculated from Oregon and Washington maps published in Data Basin (http://bit.ly/2CcZ7wK), density values are maxima of mean trends (maxima of maximum trends in parentheses) from [37] for ecoregions included in the current study; FF-WCE: full fire, with CO_2_ fertilization effect; FS-WCE: with fire suppression, with CO_2_ fertilization effect; SF-WCE: with stochastic ignitions, with CO_2_ fertilization effect; NF-WCE: no fire, with CO_2_ fertilization effect; FF-NCE: full fire, with no fertilization effect; and NF-NCE: no fire, with no CO_2_ fertilization effect)

Our simulated AGB and AGT values fall below Hudiburg’s mean trend maxima for those ecoregions in our study area (Coast Range, West Cascades, and Klamath mountains; Table 3). Our simulated AGD values fall between Hudiburg’s lowest and highest mean trend maxima and below their largest mean trend maxima (Table 3).

**Fig 6:**
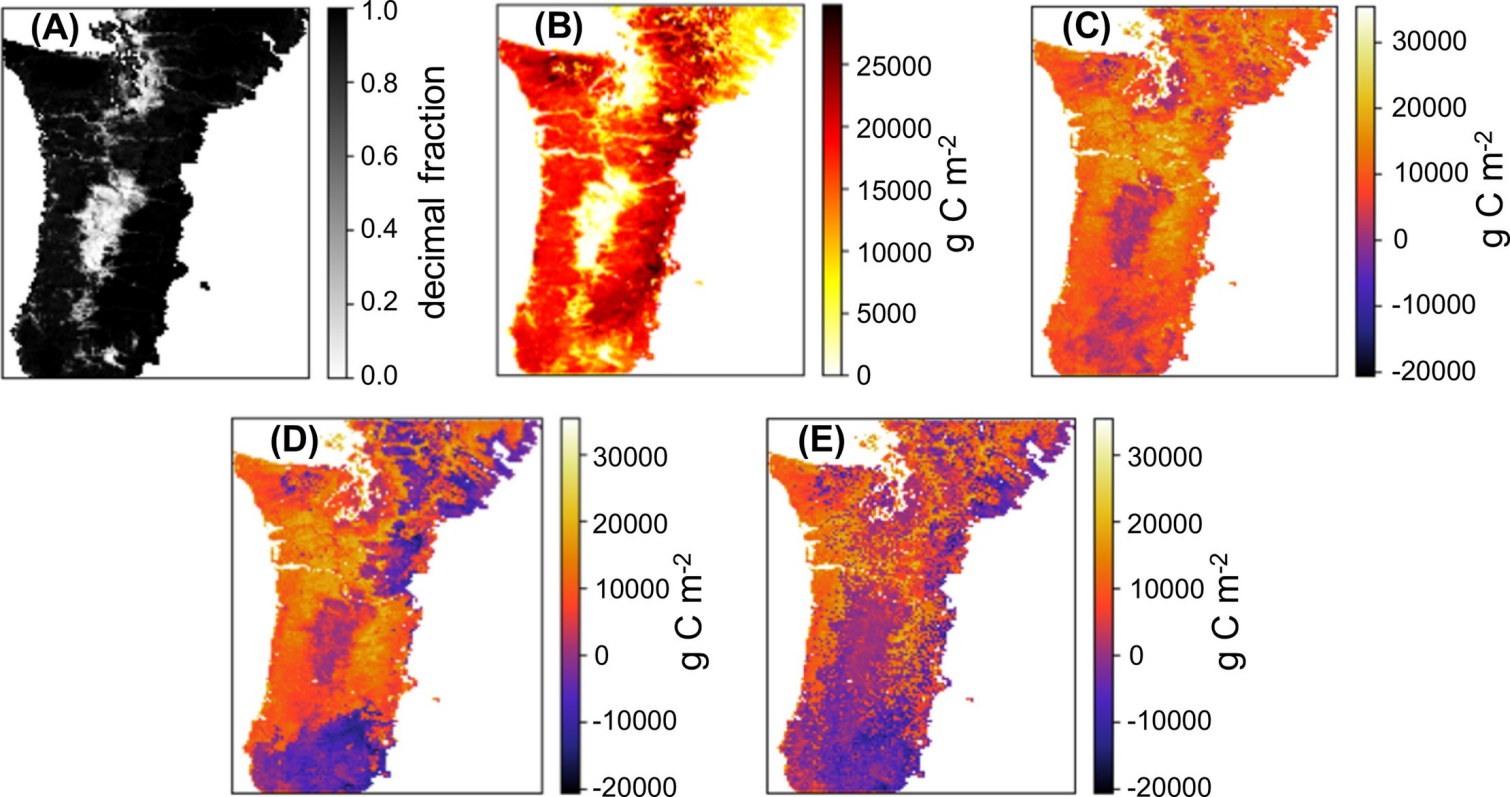
Human affected areas and carbon measures over the study area. (A) Density of NHA (non-human-affected) area used to calculate carbon densities. (B) AGT (aboveground total woody biomass) densities derived from [37] for 1991–1999. (C-E) Differences in carbon densities calculated by subtracting Hudiburg’s results from simulation results for 1991–1999: (C) NF-WCE (no fire, with CO_2_ fertilization effect); (D) FF-WCE (full fire, with CO_2_ fertilization effect) (E) SF-WCE (stochastic fire, with CO_2_ fertilization effect).

Our simulated AGT carbon density (Fig 6C–6E) is lowest in highly human affected (HA) areas (Fig 6A) such as in the Willamette Valley and surrounding Puget Sound. In these areas, there is little difference among values for our simulations or between our simulations and Hudiburg’s (Fig 6B).

For NF-WCE (Fig 6C) our simulated AGT carbon density is higher than Hudiburg’s over most of the study area. For FF-WCE (Fig 6D) our simulated AGT carbon density is generally lower in areas that have experienced fire and higher in areas that have not. Similarly, for SF-WCE, our simulated carbon density is lower in areas having experienced fire, but those areas are greater due to the spatially broader simulated fire occurrence (Fig 6D).

Compared to other studies [38, 14, 39] in the same region, our values for NPP, NEP, and NBP are generally lower, while our values for carbon stocks are higher (Table 4).

**Table 4:** Carbon flux and pool values from other studies and the current study.

**Source**	**Period**	**Method**	**NPP** **(gCm**^**-2**^**yr**^**-1**^**)**	**NEP** **(gCm**^**-2**^**yr**^**-1**^**)**	**NBP or NECB** **(gCm**^**-2**^**yr**^**-1**^**)**	**C stocks (gCm**^**-2**^**)**	**Comments**
[38]^1^	1986–2010	Biome BGC informed by field and remote sensing observations		~100 to ~200			Includes harvest, fire, and land cover
[14]^2^	1986–2010	Biome BGC informed by field and remote sensing observations	~600 to ~800	~0 to ~-600	~22 to ~67		West Cascades ecoregion only; study includes fire, timber harvest, land use, and pests
[39]^3^	1980–1997	Biome BGC informed by field and remote sensing observations	640 to 700	190 to 226	1684	32810 to 38810	Forested areas within western OR; includes fire and harvest
[37]^5^	1991–1999	Biome BGC informed by field observations	540 to 820 (~1200 to ~1500)			32810 to 46800 (~58000 to 84000)	OR and Northern CA, C Stocks are for above ground live and dead carbon including only trees and shrubs.
FF-WCE	1971–2000		1198	52	19	54400	
FS-WCE	1971–2000		1198	52	21	54500	
SF-WCE	1971–2000		1199	65	20	50900	
NF-WCE	1971–2000		1214	29	29	59700	
FF-NCE	1971–2000		1136	19	-13	53300	
NF-NCE	1971–2000		1152	-2	-3	58500	

(FF-WCE: full fire, with CO_2_ fertilization effect; FS-WCE: with fire suppression, with CO_2_ fertilization effect; SF-WCE: with stochastic ignitions, with CO_2_ fertilization effect; NF-WCE: no fire, with CO_2_ fertilization effect; FF-NCE: full fire, with no fertilization effect; and NF-NCE: no fire, with no CO_2_ fertilization effect. ^1^NEP values included for only for those ecoregions within our study area; ^2^NECB listed in table, calculated as NEP minus fire emissions minus simulated harvest removals; ^3^Values included for only forested lands in areas falling within our study area; ^4^Value is for all of forested western Oregon, reported as NBP; ^5^Oregon and northern California, values are the maximum versus stand age, values are from central trend line of data with maximum values in parentheses).

### Fire

**Fig 7:**
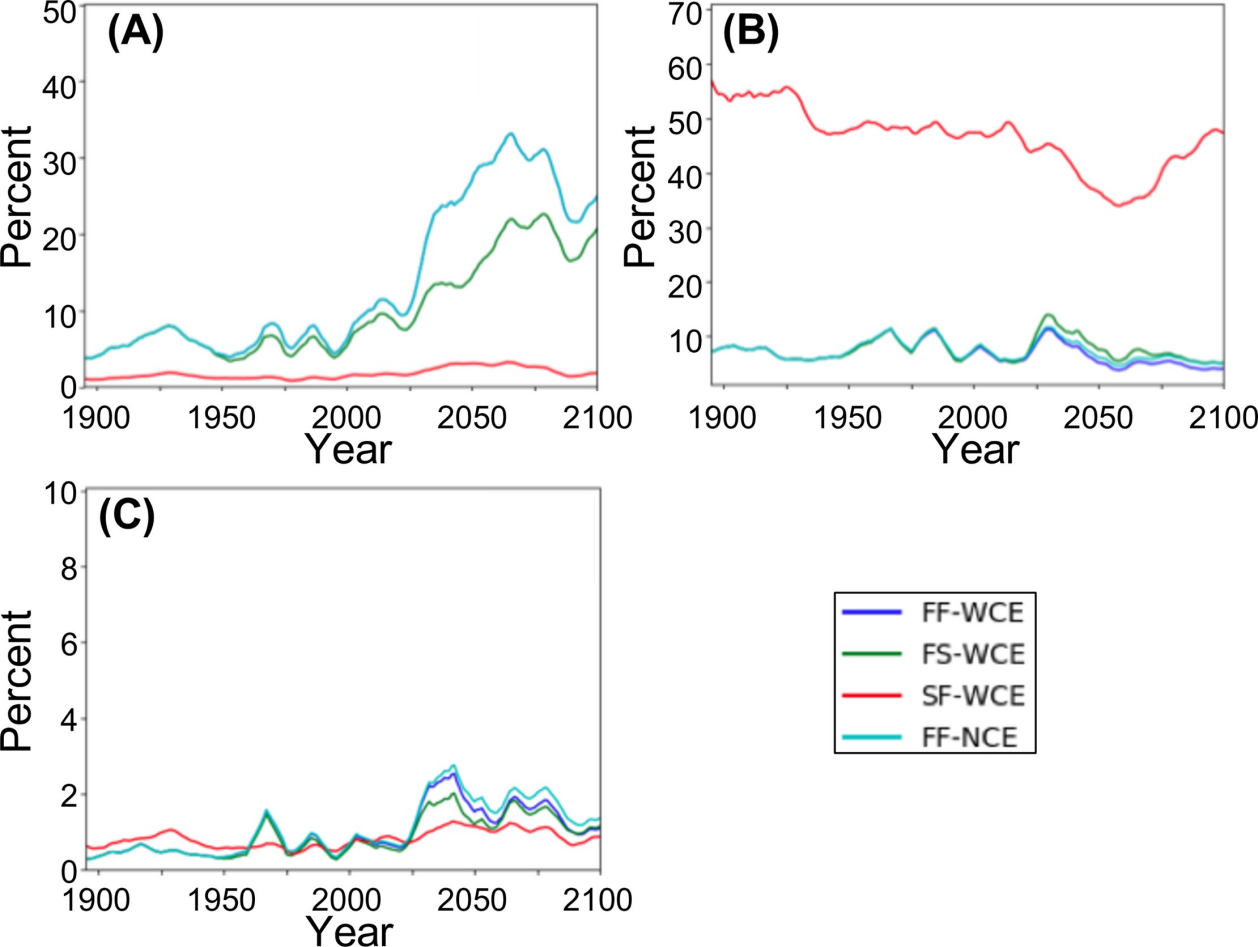
Fire results by scenario as a percentage of total area. (A) AWF (Area with fire; the area of all grid cells experiencing any fire); (B) FAB (Fraction area burned; the fraction of are burned in grid cells with fire); and (C) TAB (Total area burned; the sum of (AWF * FAB) over all grid cells). (FF-WCE: full fire, with CO_2_ fertilization effect; FS-WCE: with fire suppression, with CO_2_ fertilization effect; SF-WCE: with stochastic ignitions, with CO_2_ fertilization effect; and FF-NCE: full fire, with no fertilization effect. Results smoothed using triangle smoothing +/- 8 years).

AWF is identical for FF-NCE and FF-WCE throughout the simulation (Fig 7A, Table 5). FAB for FF-NCE is very similar to that for FF-WCE during the early 20^th^ c. but is higher through the rest of the simulation with a peak difference of 1.08% of cell area during the late 21^st^ c. (Fig 7B, Table 5). TAB for FF-NCE is similar to that for FF-WCE through the 20^th^ c., but is higher during the 21^st^ c. with a maximum difference of 0.28% during the late 21^st^ c. (Fig 7C, Table 5).

**Table 5:** Summaries of fire characteristics over the study area.

**1895–1924**	**AWF (%)**	**FAB (%)**	**TAB (%)**
**FF-WCE**	5.31	8.95	0.48
**FS-WCE**	5.31	8.95	0.48
**SF-WCE**	1.25	58.66	0.73
**FF-NCE**	5.31	8.95	0.48
**1936–1965**	**AWF (%)**	**FAB (%)**	**TAB (%)**
**FF-WCE**	4.62	8.12	0.38
**FS-WCE**	4.27	7.99	0.34
**SF-WCE**	1.21	49.44	0.60
**FF-NCE**	4.62	8.19	0.38
**1971–2000**	**AWF (%)**	**FAB (%)**	**TAB (%)**
**FF-WCE**	5.94	9.43	0.56
**FS-WCE**	4.88	9.70	0.47
**SF-WCE**	1.08	49.46	0.53
**FF-NCE**	5.94	9.64	0.57
**2036–2065**	**AWF (%)**	**FAB (%)**	**TAB (%)**
**FF-WCE**	26.93	6.47	1.74
**FS-WCE**	15.65	9.26	1.45
**SF-WCE**	2.97	38.26	1.14
**FF-NCE**	26.93	7.38	1.99
**2071–2100**	**AWF (%)**	**FAB (%)**	**TAB (%)**
**FF-WCE**	26.36	5.03	1.33
**FS-WCE**	19.90	6.37	1.27
**SF-WCE**	2.02	43.88	0.89
**FF-NCE**	26.36	6.11	1.61

Mean annual percentage of AWF (area with fire; area of gridcells in which fire occurred); FAB (fraction area burned; area weighted mean of the fraction of burning in burned grid cells); and TAB (total area burned; AWF * FAB). (FF-WCE: full fire, with CO_2_ fertilization effect; FS-WCE: with fire suppression, with CO_2_ fertilization effect; SF-WCE: with stochastic ignitions, with CO_2_ fertilization effect; and FF-NCE: full fire, with no fertilization effect)

AWF for FS-WCE is virtually identical to that for FF-WCE during the early 20^th^ c. but is less during the remainder of the simulation with the largest difference (11.28% of area) during the mid 21^st^ c. (Fig 7A, Table 5). FAB for FS-WCE is similar to that for FF-WCE through the 20^th^ c., but is greater during the 21^st^ c, with the largest difference (2.79% of cell area) during the mid 21^st^ c. (Fig 7B, Table 5). TAB for FS-WCE is identical to that for FF-WCE during the early 20^th^ c., but lower during all other periods, with the largest difference (0.29% of area) during the mid 21^st^ c. (Fig 7C, Table 5).

AWF is consistently lower for SF-WCE than for FF-WCE during the entire simulation with the greatest difference (24.34% of area) during the late 21^st^ c. (Fig 7A, Table 5). FAB is consistently higher for SF-WCE than for FF-WCE throughout the simulation with the greatest difference (49.71% of area) occurring in the early 20^th^ c. (Fig 7B, Table 5). TAB is initially higher for SF-WCE than for FF-WCE during the early and mid 20^th^ c. (largest difference of 0.28% of area during mid 20^th^ c.) but is lower for the remainder of the simulation (largest difference of 0.60% of area during the mid 21^st^ c.; Fig 7B, Table 5).

### Carbon fluxes

**Fig 8:**
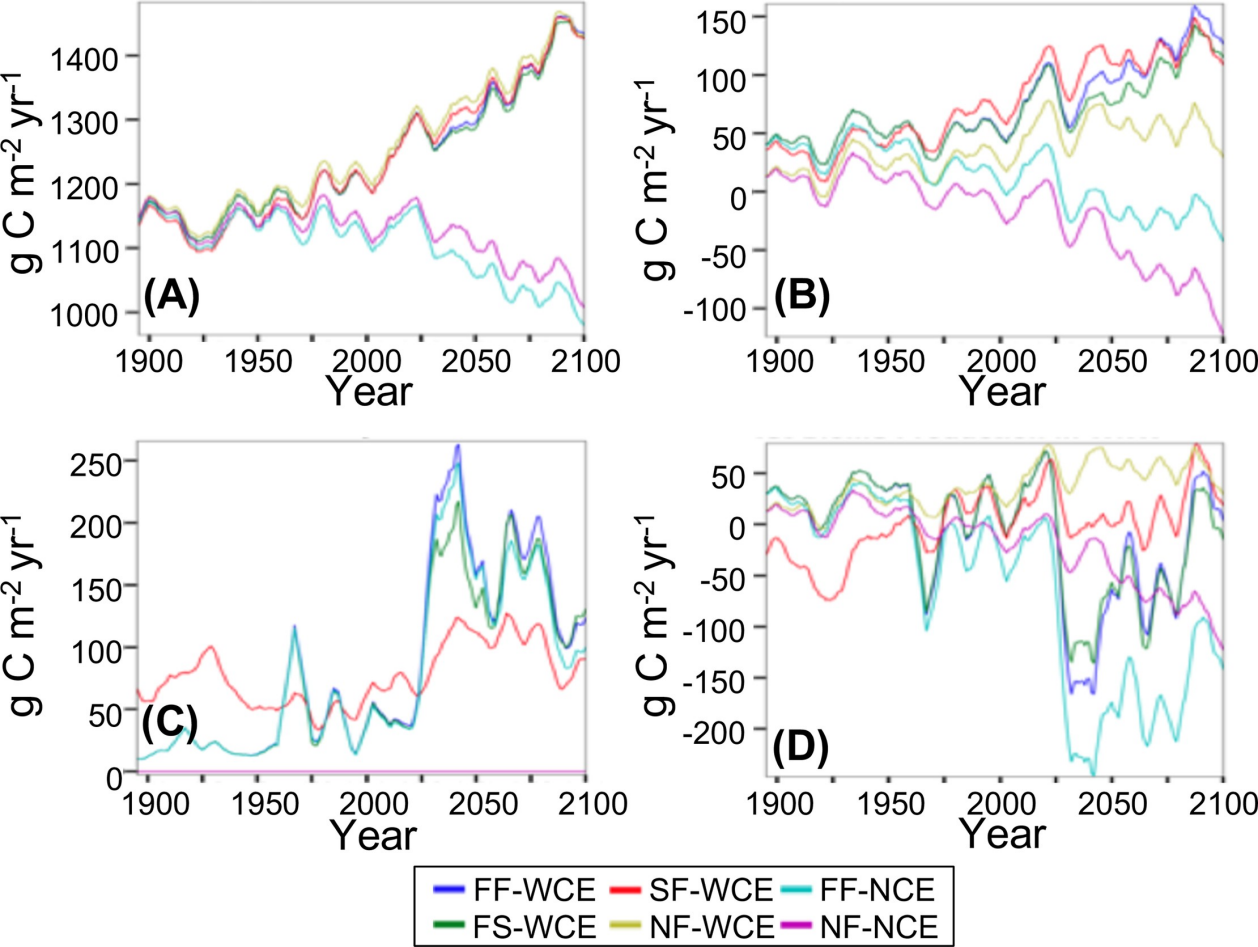
Carbon fluxes simulated by the MC2 vegetation model. (A) NPP (net primary production), (B) NEP (net ecosystem production), (C) C consumed by fire, and (D) NBP (net biome production). (FF-WCE: full fire, with CO_2_ fertilization effect; FS-WCE: with fire suppression, with CO_2_ fertilization effect; SF-WCE: with stochastic ignitions, with CO_2_ fertilization effect; NF-WCE: no fire, with CO_2_ fertilization effect; FF-NCE: full fire, with no fertilization effect; and NF-NCE: no fire, with no CO_2_ fertilization effect. Results smoothed using triangle smoothing +/- 8 years).

For WCE scenarios, NPP increases by approximately 5% over the 20^th^ c. and an additional 18% over the 21^st^ c. (Fig 8A, Table 6). NPP varies by less than 3% across all WCE scenarios within any time period. For FF-NCE, NPP does not vary over the 20^th^ c. but decreases by 10% over the 21^st^ c. (Fig 8A, Table 6). NPP for NF-NCE increases by 1% over the 20^th^ c. and decreases by 8% over the 21^st^ c. (Fig 8A, Table 6).

**Table 6:** Mean (standard deviation in parentheses) carbon fluxes by time period for simulation scenarios.

**NPP (g C m**^**-2**^ **yr**^**-1**^**)**					
	**1895–1924**	**1936–1965**	**1971–2000**	**2036–2065**	**2071–2100**
**FF-WCE**	1143 (79)	1173 (94)	1198 (87)	1315 (132)	1422 (136)
**FS-WCE**	1143 (79)	1173 (94)	1198 (87)	1307 (130)	1413 (134)
**SF-WCE**	1131 (79)	1156 (92)	1199 (87)	1329 (133)	1420 (133)
**NF-WCE**	1150 (80)	1179 (94)	1214 (87)	1344 (131)	1430 (130)
**FF-NCE**	1136 (80)	1149 (93)	1136 (81)	1072 (122)	1024 (109)
**NF-NCE**	1143 (80)	1155 (93)	1152 (81)	1111 (126)	1060 (114)
**NEP (g C m**^**-2**^ **yr**^**-1**^**)**					
	**1895–1924**	**1936–1965**	**1971–2000**	**2036–2065**	**2071–2100**
**FF-WCE**	37 (46)	55 (50)	52 (49)	99 (66)	136 (100)
**FS-WCE**	37 (46)	55 (50)	52 (49)	82 (63)	121 (97)
**SF-WCE**	27 (46)	48 (49)	65 (50)	115 (67)	126 (96)
**NF-WCE**	10 (46)	27 (49)	29 (49)	62 (62)	54 (92)
**FF-NCE**	32 (46)	42 (49)	19 (44)	-12 (61)	-19 (76)
**NF-NCE**	5 (46)	14 (48)	-3 (44)	-40 (63)	-82 (80)
**Consumed (g C m**^**-2**^ **yr**^**-1**^**)**					
	**1895–1924**	**1936–1965**	**1971–2000**	**2036–2065**	**2071–2100**
**FF-WCE**	20 (32)	15 (19)	34 (55)	177 (217)	145 (139)
**FS-WCE**	20 (32)	15 (18)	31 (52)	155 (199)	139 (132)
**SF-WCE**	71 (51)	53 (36)	45 (30)	112 (72)	92 (48)
**NF-WCE**	0 (0)	0 (0)	0 (0)	0 (0)	0 (0)
**FF-NCE**	20 (32)	15 (18)	33 (54)	169 (200)	126 (124)
**NF-NCE**	0 (0)	0 (0)	0 (0)	0 (0)	0 (0)
**NBP (g C m**^**-2**^ **yr**^**-1**^**)**					
	**1895–1924**	**1936–1965**	**1971–2000**	**2036–2065**	**2071–2100**
**FF-WCE**	17 (61)	40 (56)	19 (87)	-77 (257)	-7.9 (214)
**FS-WCE**	17 (61)	40 (56)	21 (85)	-73 (238)	-18 (207)
**SF-WCE**	-44 (71)	-5 (70)	20 (64)	3 (126)	34 (122)
**NF-WCE**	10 (46)	27 (49)	29 (49)	62 (62)	54 (92)
**FF-NCE**	12 (61)	27 (55)	-13 (81)	-181 (233)	-144 (174)
**NF-NCE**	5 (46)	14 (48)	-3 (43)	-40 (63)	-82 (80)

(NPP: net primary production; NEP: net ecosystem production; NBP: net biome production; FF-WCE: full fire, with CO_2_ fertilization effect; FS-WCE: with fire suppression, with CO_2_ fertilization effect; SF-WCE: with stochastic ignitions, with CO_2_ fertilization effect; NF-WCE: no fire, with CO_2_ fertilization effect; FF-NCE: full fire, with no fertilization effect; and NF-NCE: no fire, with no CO_2_ fertilization effect)

Over the simulation period, NEP increases for WCE scenarios and decreases for both NCE scenarios (Fig 8B, Table 6). Throughout the simulation period, for FF-WCE and FF-NCE, NEP increases more than for their no-fire counterparts (Fig 8B, Table 6).

Consumed C for with-fire scenarios (FF-WCE, FS-WCE, FF-NCE) is nearly identical during the 20^th^ c. (Fig 8C, Table 6). For these scenarios, consumed C triples from the late 20^th^ c. to the late 21^st^ c. The pattern of consumed C is similar among these scenarios throughout the 21^st^ c., but the range of values increases by the end of the 21^st^ c. For SF-WCE, consumed C is higher than that for FF-WCE over the 20^th^ c. but lower during the 21^st^ c. The standard deviation of SF-WCE consumed C is higher than that for FF-WCE during the early and mid 20^th^ c. but lower during the late 20^th^ c. and the mid 21^st^ c. (Table 6).

During the early and mid 20^th^ c. NBP is lower for SF-WCE than that for all other scenarios but becomes higher than that for all scenarios except NF-WCE by the end of the 21^st^ c. (Fig 8D, Table 6). NBP for SF-WCE shows much less variability than FF-WCE in the mid and late 21^st^ c. NBP for FF-WCE and FS-WCE falls sharply from the late 20^th^ c. to the mid 21^st^ c. but rises during the late 21^st^ c. NBP for FF-NCE decreases more abruptly from the late 20^th^ c. to the mid 21^st^ c. and increases less during the late 21^st^ c. NBP for NF-WCE increases from the late 20^th^ to the mid 21^st^ c. then decreases during the late 21^st^ c. NBP for NF-NCE decreases from the late 20^th^ c. through the mid and late 21^st^ c.

### Carbon pools

**Fig 9:**
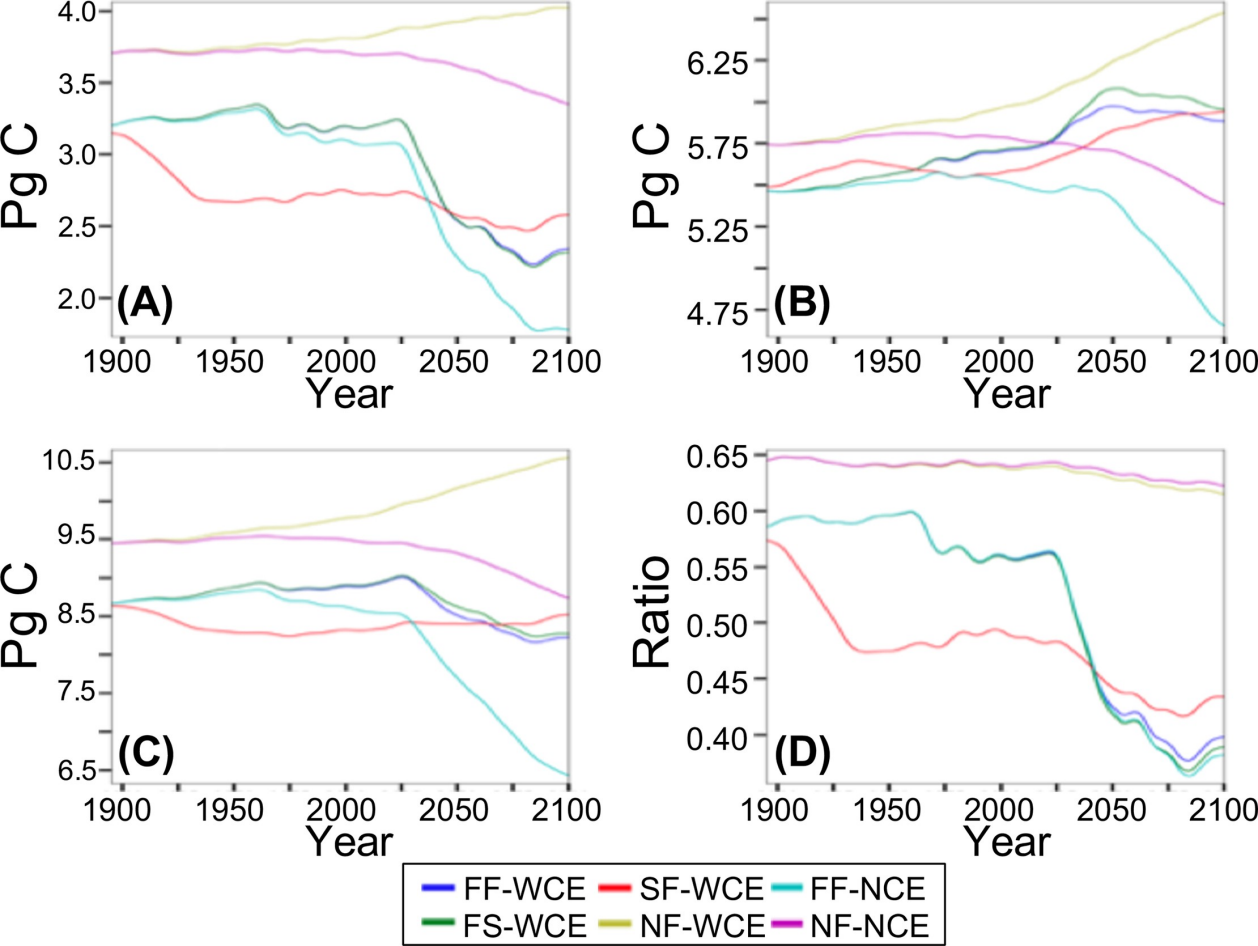
Carbon pools and live/dead ratios. (A) live C, (B) dead C, (C) ecosystem C, and (D) ratio of live C to dead C. (FF-WCE: full fire, with CO_2_ fertilization effect; FS-WCE: with fire suppression, with CO_2_ fertilization effect; SF-WCE: with stochastic ignitions, with CO_2_ fertilization effect; NF-WCE: no fire, with CO_2_ fertilization effect; FF-NCE: full fire, with no fertilization effect; and NF-NCE: no fire, with no CO_2_ fertilization effect. Results smoothed using triangle smoothing +/- 8 years).

Live C (C in live plants), dead C (standing dead trees, litter, and soil C), and total ecosystem C (ecosystem C hereafter) for NF-WCE increase throughout the simulation by 4, 6, and 10% respectively, with the live to dead C ratio decreasing from 0.65 to 0.61 (Fig 9A–9D, Table 7). For FF-WCE and FS-WCE live C decreases 7.5% from the late 20^th^ c. to the late 21^st^ c. (Fig 9A, Table 7). Over the same period dead C pools increase 4 and 6% for FF-WCE and FS-WCE respectively, and ecosystem C decreases 7% for both scenarios, and live to dead ratios decrease from 0.56 to 0.39 and 0.56 to 0.38 for FF-WCE and NF-WCE respectively (Fig 9A–9D, Table 7). For FF-NCE, live, dead C, and ecosystem C decrease by 41, 12, and 22% respectively from the late 20^th^ c. through the late 21^st^ c. and the live to dead C ratio decreases (from 0.56 to 0.38; Fig 9A–9D, Table 7).

**Table 7:** Mean (standard deviation in parentheses) carbon pool values and live to dead C ratios by time period.

**Live Carbon (Pg)**					
	**1895–1924**	**1936–1965**	**1971–2000**	**2036–2065**	**2071–2100**
**FF-WCE**	3.24 (0.025)	3.32 (0.033)	3.18 (0.032)	2.60 (0.149)	2.30 (0.059)
**FS-WCE**	3.24 (0.025)	3.32 (0.033)	3.18 (0.032)	2.60 (0.151)	2.28 (0.058)
**SF-WCE**	3.03 (0.104)	2.66 (0.017)	2.72 (0.037)	2.59 (0.053)	2.52 (0.048)
**NF-WCE**	3.72 (0.014)	3.75 (0.019)	3.79 (0.020)	3.93 (0.025)	4.00 (0.024)
**FF-NCE**	3.23 (0.024)	3.29 (0.027)	3.12 (0.036)	2.33 (0.186)	1.83 (0.078)
**NF-NCE**	3.72 (0.014)	3.72 (0.014)	3.72 (0.011)	3.61 (0.037)	3.43 (0.053)
**Dead Carbon (Pg)**					
	**1895–1924**	**1936–1965**	**1971–2000**	**2036–2065**	**2071–2100**
**FF-WCE**	5.47 (0.015)	5.56 (0.019)	5.67 (0.024)	5.95 (0.037)	5.91 (0.024)
**FS-WCE**	5.47 (0.015)	5.56 (0.019)	5.68 (0.026)	6.04 (0.050)	6.00 (0.033)
**SF-WCE**	5.54 (0.042)	5.62 (0.022)	5.56 (0.015)	5.81 (0.049)	5.92 (0.015)
**NF-WCE**	5.75 (0.014)	5.85 (0.018)	5.92 (0.027)	6.24 (0.063)	6.46 (0.052)
**FF-NCE**	5.47 (0.012)	5.52 (0.009)	5.55 (0.020)	5.38 (0.100)	4.87 (0.150)
**NF-NCE**	5.75 (0.011)	5.80 (0.008)	5.79 (0.010)	5.67 (0.030)	5.48 (0.069)
**Total Ecosystem Carbon (Pg)**					
	**1895–1924**	**1936–1965**	**1971–2000**	**2036–2065**	**2071–2100**
**FF-WCE**	8.70 (0.028)	8.88 (0.049)	8.85 (0.026)	8.54 (0.124)	8.21 (0.052)
**FS-WCE**	8.71 (0.028)	8.88 (0.050)	8.87 (0.027)	8.64 (0.110)	8.28 (0.052)
**SF-WCE**	8.57 (0.064)	8.29 (0.016)	8.28 (0.032)	8.40 (0.017)	8.44 (0.054)
**NF-WCE**	9.47 (0.017)	9.60 (0.033)	9.71 (0.042)	10.2 (0.083)	10.5 (0.072)
**FF-NCE**	8.70 (0.023)	8.81 (0.032)	8.67 (0.033)	7.71 (0.263)	6.69 (0.213)
**NF-NCE**	9.46 (0.013)	9.52 (0.016)	9.51 (0.007)	9.30 (0.063)	8.91 (0.120)
**Live / Dead (ratio)**					
	**1895–1924**	**1936–1965**	**1971–2000**	**2036–2065**	**2071–2100**
**FF-WCE**	0.59 (0.005)	0.60 (0.004)	0.56 (0.007)	0.44 (0.027)	0.39 (0.011)
**FS-WCE**	0.59 (0.005)	0.60 (0.004)	0.56 (0.008)	0.43 (0.028)	0.38 (0.011)
**SF-WCE**	0.55 (0.023)	0.48 (0.005)	0.49 (0.008)	0.45 (0.013)	0.42 (0.008)
**NF-WCE**	0.65 (0.003)	0.64 (0.003)	0.64 (0.003)	0.63 (0.004)	0.61 (0.003)
**FF-NCE**	0.59 (0.005)	0.60 (0.005)	0.56 (0.007)	0.43 (0.030)	0.38 (0.011)
**NF-NCE**	0.65 (0.003)	0.64 (0.003)	0.64 (0.003)	0.64 (0.005)	0.63 (0.003)

(Pg: petagram; FF-WCE: full fire, with CO_2_ fertilization effect; FS-WCE: with fire suppression, with CO_2_ fertilization effect; SF-WCE: with stochastic ignitions, with CO_2_ fertilization effect; NF-WCE: no fire, with CO_2_ fertilization effect; FF-NCE: full fire, with no fertilization effect; and NF-NCE: no fire, with no CO_2_ fertilization effect)

For SF-WCE, live C decreases by 16% from the early 20^th^ c. to the mid 20^th^ c., dead C increases 7% from the late 20^th^ to the late 21^st^ c., and ecosystem C decreases by 2% from the early 20^th^ to the late 21^st^ c. (Fig 9A–9C, Table 7). Over the same period, the live to dead C ratio decreases from 0.55 to 0.42 (Fig 9D, Table 7).

### Vegetation

**Fig 10:**
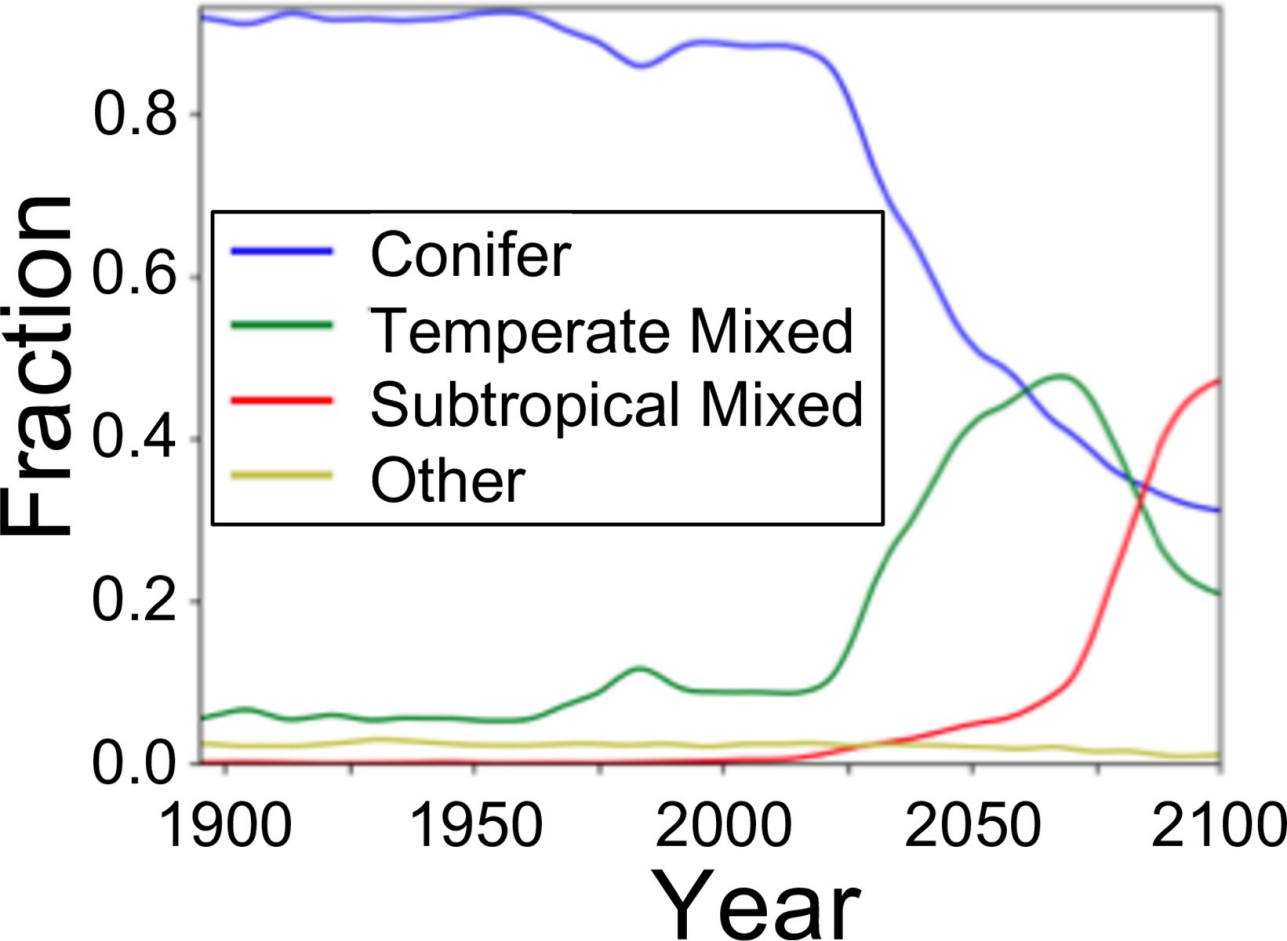
Vegetation class mix over time for FF-WCE (full fire with CO_2_ fertilization) scenario. All other scenarios yield virtually identical results.

Vegetation composition is consistent across all scenarios, with the differences of 3% or less for all categories in each time period (Fig 10, Table 8). The *other* category (non-forest) comprises two percent or less of the area from the early 20^th^ c. through the late 21^st^ c. Through the 20^th^ c., conifer covers between 88% to 94% of the area with temperate mixed forest accounting for the remainder of the forested area. From the late 20^th^ to mid 21^st^ c. conifer forest extent decreases to 53 to 55% of the area, temperate mixed forest increases to 39 to 40%, and subtropical mixed forest increases to 5% of the area. During the late 21^st^ c. conifer forest extent decreases to 34 to 35%, temperate mixed forest decreases to 32%, and subtropical mixed forest increases to 33% of the area.

**Table 8:** Simulated vegetation composition (%) of study area.

**1895–1924**	**Con**	**T Mix**	**S Mix**	**Oth**
**FF-WCE**	92	6	0	2
**FS-WCE**	92	6	0	2
**SF-WCE**	92	6	0	2
**NF-WCE**	94	6	0	0
**FF-NCE**	92	6	0	2
**NF-NCE**	94	6	0	0
**1936–1965**	**Con**	**T Mix**	**S Mix**	**Oth**
**FF-WCE**	93	5	0	2
**FS-WCE**	93	5	0	1
**SF-WCE**	92	5	0	2
**NF-WCE**	94	5	0	0
**FF-NCE**	93	5	0	2
**NF-NCE**	94	5	0	0
**1971–2000**	**Con**	**T Mix**	**S Mix**	**Oth**
**FF-WCE**	88	10	0	2
**FS-WCE**	88	10	0	2
**SF-WCE**	88	10	0	2
**NF-WCE**	90	10	0	0
**FF-NCE**	87	10	0	3
**NF-NCE**	90	10	0	0
**2036–2065**	**Con**	**T Mix**	**S Mix**	**Oth**
**FF-WCE**	54	40	5	2
**FS-WCE**	53	40	5	3
**SF-WCE**	53	40	5	2
**NF-WCE**	55	40	5	0
**FF-NCE**	53	39	5	3
**NF-NCE**	55	40	5	0
**2071–2100**	**Con**	**T Mix**	**S Mix**	**Oth**
**FF-WCE**	35	32	33	0
**FS-WCE**	34	32	33	1
**SF-WCE**	34	32	33	1
**NF-WCE**	35	32	33	0
**FF-NCE**	34	32	33	1
**NF-NCE**	35	32	33	0

(Con: conifer; T Mix: temperate mixed conifer and broadleaf; S Mix: subtropical mixed conifer and broadleaf; Oth: other; Veg: vegetation; FF-WCE: full fire, with CO_2_ fertilization effect; FS-WCE: with fire suppression, with CO_2_ fertilization effect; SF-WCE: with stochastic ignitions, with CO_2_ fertilization effect; NF-WCE: no fire, with CO_2_ fertilization effect; FF-NCE: full fire, with no fertilization effect; and NF-NCE: no fire, with no CO_2_ fertilization effect)

## Discussion

### Validation, comparison with other studies, and limitations

For unlimited ignition scenarios versus observed fires there is general agreement between areas where area burned is greatest. The lack of concentrated modeled TAB in the southwest corner of the region versus observed (Fig 4C vs 4B) may be due to the use of a dataset with deeper soils than are actually present. In the model, shallower soils retain less water, potentially leading to drier fuel conditions and greater fire. In this part of the study area, the STATSGO dataset used in the simulation has soil depths two to four times as deep as the more recent SSURGO dataset [40].

The overall higher simulated TAB and the higher simulated TAB east of Puget Sound–primarily due to simulated fires in 1987 and 2003 –underscore the importance of modeling wildfire ignition limitations in addition to fuel limitations. Stochastic fire (SF) mitigates the overall higher TAB, but simulates more fires than observed in areas that are commonly fuel limited (Cascades and Coast range). The stochastic ignitions algorithm used in the SF-WCE scenario was implemented as a proof of concept with a random algorithm to locate ignition sources. An algorithm using a probability surface based on factors affecting ignition sources such as human presence and infrastructure (e.g. [41–42]) and lightning strikes (e.g. [43–44]) would likely produce more realistic results but this kind of data is lacking both for the beginning of 20^th^ century and for the 21^st^ century. Secondly, informing the algorithm with known relationships between fuel conditions and fire initiation would likely also improve results. Additionally, considering conditions more or less conducive to fire initiation, such as slope and other topographic characteristics could also contribute to higher quality fire modeling. Multiple model runs with stochastic ignition sources and success should also be considered to generate a statistics-based projection of fire on the landscape.

Differences between our results and those of other studies (Table 4) are due to a number of factors. First, observation-based models generally only consider aboveground stocks, while MC2 models above- and belowground carbon, including soils and litter. Second, other studies include disturbances that MC2 does not, for example logging, insect infestations, and disease, which would account for some of the higher carbon stock value in our results. Third, the mapping of human-affected (HA) areas reduces differences due to land use, but HA areas can only be considered a first approximation of human influences on the landscape, including historical logging in areas now recovering and fires not accounted for in the simulation, (e.g. the Tillamook fire; [45]).

Our results contrast with [46], which projects increasing forest carbon stocks in Oregon forests through the 21^st^ c. That study assumes a CO_2_ fertilization effect. However, unlike ours, that study includes mortality from forest harvest and beetles and also assumes the same vegetation that is lost regrows at its maximum potential.

MC2 simulates potential vegetation most adapted to climate inputs. However, vegetation can endure under suboptimal conditions, slowing the replacement of one vegetation type by another, remaining until a sudden disturbance or mortality due to crossing a physiological threshold allows for rapid change. Moreover, MC2 does not simulate seed production, seedling establishment, or natural succession. These factors should be accounted for when using model projections for management decisions.

Land use, insects, pathogens, and invasive species are important disturbances that may be amplified or mitigated by climate change (e.g. [47–49]). Including them in the DGVM is desirable, but would require a better understanding of a wide variety of pests’ and invasive species’ response to climate change as well as calibration datasets that are still often lacking.

Soil data are critical for projecting accurate soil water availability and drought stress [40]. More accurate soil data would improve the reliability of growth, mortality, and fire simulation results. More recent MC2 simulations have used SSURGO data but at the time of this project, the dataset was still incomplete for large portions of our study area.

### Effects of model assumptions on vegetation

The simulated transition from conifer to temperate mixed conifer/broadleaf to subtropical mixed conifer/broadleaf takes place at a similar rate and with a similar pattern regardless of fire and CO_2_ fertilization and can be attributed solely to climate change. Other studies using MC2 and its predecessor, MC1, project vegetation shifts in this region towards warmer and mixed forests [1–2, 14, 50], however, to our knowledge, ours is the first study to show that this shift is purely climate driven. This result stands in contrast to the simulated fire regime-driven vegetation shifts in other regions found in other studies using MC2 (e.g. [1, 31])

### Effects of model assumptions on fire

With or without fire suppression, unlimited ignitions cause a sharp increase in AWF as climate conditions drive fuel conditions over ignition thresholds on a yearly basis during the early and mid 21^st^ c. This is consistent with recent observed increases in wildfire across the western US, including the PNW, due to warming climate [3, 51] and specifically in the Western Cascades due to decreased May through September precipitation [52]. The initial decrease in FAB and TAB as AWF remains high is due to the dependency of FAB on the combination of time since last fire and fire return interval (FRI) in addition to fuel conditions. The longer a cell does not burn, the greater the fraction of that cell that can burn. With the initial transition to more frequent fires, the first fire in a cell burns a greater cell portion than subsequent fires in the same cell. The vegetation shift to subtropical mixed forest, which has a higher ignition threshold, contributes to decreased AWF, FAB, and TAB towards the end of the 21^st^ c. The higher fuel thresholds of fire suppression reduce AWF and TAB due to fuel conditions reaching ignition thresholds less frequently. Less frequent fires, however, account for the greater TAB under fire suppression.

Stochastic fire responds to the same drivers as the unlimited ignitions but in different ways. First, the use of an ignition probability function instead of a single fuel threshold allows fires to occur even under fuel conditions less severe than those at unlimited ignition threshold levels. Thus, during the early 20^th^ c. some cells not experiencing fire under unlimited ignitions do experience fire under stochastic ignitions leading to a greater TAB under stochastic fire. Second, the probabilistic nature of ignition sources and fire initiation limits fire occurrence under fuel conditions exceeding thresholds, as during the early to late 21^st^ c. when fire occurrence is more common across the entire study area under unlimited ignitions than under stochastic fire. During this period, TAB is lower under stochastic fire than under unlimited ignitions scenarios due to less frequent fire occurrence. The overall less frequent fires due to stochastic fire result in a lower AWF and higher FAB. Stochastic fire occurring below the thresholds set for unlimited ignitions accounts for the higher FAB at the end of the 21^st^ c., when stochastic fire initiates fires in subtropical mixed forest while unlimited ignitions does not.

Fire effects due to CO_2_ fertilization assumptions are generally small. However, even though AWF is identical for FF-NCE and FF-WCE, FAB and TAB are slightly higher for FF-NCE. We attribute this to dryer fuel conditions as a result of lower water use efficiency (WUE) for FF-NCE.

### Effects of model assumptions on carbon

Separate from fire, CO_2_ fertilization under climate change increases productivity and C in all pools through time. Conversely the lack of CO_2_ fertilization under climate change decreases productivity C in all pools. The smooth changes in C pools and ratio of live to dead C for both no-fire scenarios indicate little change in carbon dynamics. The continuing increases in all C pools for NF-WCE and decreases for NF-NCE indicate that climate continues to influence production through the end of the 21^st^ c.

CO_2_ fertilization is the strongest driver of NPP as shown by the similar increases in NPP across all scenarios with CO_2_ fertilization versus the similar decrease for scenarios without CO_2_ fertilization. Consumed C is very similar for all scenarios with unlimited ignitions. Increased carbon storage in young forests recovering from fire as well as to the reduction of dead material available for decomposition due to burning drive higher NEP for all scenarios with CO_2_ fertilization and unlimited ignitions. However, for FF-NCE, NEP decreases due to decreasing NPP.

For scenarios with unlimited ignitions, consumed C directly reflects TAB increasing sharply in the mid 20^th^ c. and then decreasing. This pattern is further reflected in NBP which decreases and increases with TAB. By the end of the 21^st^ c., NBP is close to 0 g C m^-2^ yr^-1^ for scenarios with unlimited ignitions and with CO_2_ fertilization, indicating a possible equilibrium in carbon dynamics. However, for FF-NCE, NBP remains negative, indicating further C losses. For SF-WCE, consumed C and NBP also reflect TAB, with consumed C greater than that for unlimited-ignitions scenarios in the early 20^th^ c. and less during the mid and late 21^st^ c.

More live C is lost due to fire than is added due to CO_2_ fertilization. For unlimited ignitions, losses are greatest during the mid to late 21^st^ c. when fuel thresholds are exceeded. For stochastic fire, the greatest losses are in the early 20^th^ c. due to fires occurring where they cannot under unlimited ignitions. CO_2_ fertilization, however, may provide enough productivity to maintain a new equilibrium with increased dead C and limited decreases in ecosystem C. For all with-CO_2_ fertilization, with-fire scenarios, at the end of the 21^st^ c., the steady values of all C stocks and live to dead C ratios indicate the possibility of a new equilibrium. This is consistent with the near 0 g C m^-2^ yr^-1^ NBP for these scenarios at the end of the 21^st^ c.

The largest decreases in all C stocks are for the with-fire, without-CO_2_ fertilization. In addition, while live vegetation C becomes steady for this scenario at the end of the 21^st^ c., dead C, and ecosystem C continue to decrease, indicating that equilibrium has not been reached. This is consistent with the negative NBP for this scenario at the end of the 21^st^ c.

### Implications

The sharp, climate-driven increases in area with fire and total area burned during the first half of the 21^st^ suggest this region will be susceptible to climate-driven trend towards “large,” “very large,” or “extreme” wildfire events or fires [53] observed in the United States [54, 55], Europe [56, 57], and globally [58]. The increases also indicate that this region will experience the associated increases in infrastructure loss, suppression costs, and natural resource loss [59], and underscore the importance of understanding the effects of alternative management scenarios in fire-prone landscapes [60].

The live C lost in all with-fire scenarios indicates fire will cause mortality through the 21^st^ c. Another indication of future vegetation mortality is the climate-driven transition from needleleaf to temperate mixed to warm mixed forest. MC2 does not simulate mortality, seeding, sprouting, recruitment, and succession associated with forest type change driven by climate alone. So, for example, under a warming climate, the model could shift a forest dominated by needleleaf lifeforms to one dominated by mixed conifer and hardwood lifeforms without simulating mortality and succession. Thus, model results should be interpreted as a suggestion that vegetation will come under stress due to a changing climate. Mortality and vegetation type change would not necessarily be sudden. However, stressed vegetation would be more susceptible to disturbances [48] such as drought, fire, insects [49, 61], and disease, with the resulting mortality providing opportunity for vegetation to change from legacy to a more suited type.

Shorter FRIs combined with reduced recruitment due to changed climate conditions has the potential to extirpate species locally [62]. Furthermore, climate change velocity, especially in combination with pest outbreaks, could outpace species’ migration rates [63], leaving portions of the area depauperate. The subtropical mixed forests projected to occupy much of the area by the end of the 21^st^ c. are characterized by both needleleaf and broadleaf evergreens and would be similar to northern Californian forests which contain evergreen California live oaks (*Quercus agrifolia*). However, the decline of oak populations due to sudden oak death syndrome (*Phytophthora ramorum)* in California and Oregon [64] challenges any assumption of a northward migration of this evergreen broadleaf species.

Ecosystem C decreases in all with-fire scenarios indicate that this region will become a carbon source in the future. Decreases may be more pronounced beyond 2100 if the increased C in the dead pool decays at a greater rate than dead C is produced. This is possible as the simulated increase in dead C is due to a sudden increase in fire in forests that had been highly productive, and the live to dead C ratios increase towards previous equilibrium values at the end of the simulation. If the CO_2_ effect is lower than projected or is temporary, with plants adapting to the new CO_2_ concentrations, carbon losses may be higher than projected under the WCE scenarios, for example, 1.69 Pg C (20%) greater for the FF-NCE than for the FF-WCE. However black carbon accumulation from more frequent fire and the possible slowing of decomposition due to higher evaporative demand in soils may mitigate losses of soil C.

Under our projections, a variety of ecosystem services could be impacted. As previously stated, carbon sequestration could be reduced. Widespread mortality would reduce timber available for harvest, and rapid change of vegetation types could result in a lack of mature trees for harvest. The implied negative impacts on forests could affect fresh water supplies [65], wildlife habitat quality, and recreation.

## Conclusions

For the area west of the Cascade Crest in Oregon and Washington, we found assumptions about CO_2_ fertilization effects and fire occurrence in the MC2 DVGM have substantial effects on simulated carbon dynamics. Without fire, CO_2_ fertilization increases C stocks, while the lack of CO_2_ fertilization leads to decreases in C stocks. For scenarios with fire, CO_2_ fertilization mitigates projected C losses due to fire, limiting decreases over the 20^th^ and 21^st^ centuries by a factor of 4 versus scenarios without CO_2_ fertilization.

Stochastic fire occurrence dampens the sudden increases in area with fire, and total area burned simulated under unlimited ignitions. As a result, C pools are more stable through time under stochastic fire occurrence than under unlimited ignitions. The stark differences between results for unlimited ignitions and those for stochastic fire occurrence point to the need for further research regarding fire occurrence algorithms in DGVMs. Areas for further research include: the addition of ignition source probabilities to guide the location of fire occurrence; fire spread which would allow modeling large fires across grid cells; inclusion of land use and land cover to shape both the occurrence and spread of fire; and the elimination of fire return intervals from fire algorithms in order to model fire occurrence and extent from physical parameters and stochastic events without imposed limitations.

Vegetation is projected to change from predominantly conifer to predominantly mixed conifer and hardwood forests, regardless of CO_2_ fertilization and fire effects. With climate, not fire, driving vegetation change, much of the current vegetation can be expected to experience mortality. It is reasonable to anticipate that climate stress will make forests more susceptible disease and pests, which are not modeled by MC2.

These projections underscore ongoing challenges for resource managers who must balance the possibly competing concerns of wildfire, forest condition, wildlife management, carbon sequestration, high potential for vegetation change, and a variety of ecosystem services including clean water and air. Nonetheless, this study and its conclusions should be taken in a broader context. MC2 is one of many models suitable to explore the possible futures of this region. Given the region’s ecological and economic importance, extensive monitoring is warranted to provide insight into the state of the forests, possibly confirming or refuting signs of stress, vegetation change, and ecological threshold exceedance.

## Supporting information

S1 Table Reclassifications of MC2 vegetation types. (DOCX)

S1 Fig Selected climate results for this study. (A) CCSM4 RCP 8.5 annual and April-September maximum temperature, (B) CCSM4 RCP 8.5 annual and April-September precipitation, and (C) Annual PET calculated by MC2. All results smoothed using triangle smoothing +/- 8 years.
(PDF)

S2 Table Mean (standard deviation in parentheses) over study region for selected climate variables by time period. (DOCX)
