# Supplementary material for: Fire, CO2, and climate effects on modeled vegetation and carbon dynamics in western Oregon and Washington
Source: PLoS One. 2019 Jan 25;14(1):e0210989. doi: 10.1371/journal.pone.0210989 (PMC6347276; doi:10.1371/journal.pone.0210989)
Supplement: S1 Table — (DOCX) [file pone.0210989.s001.docx]

| **Reclassified vegetation type** | **MC2 vegetation types** |
| --- | --- |
| Conifer forest | Subalpine, Maritime Evergreen Needleleaf Forest, Temperate Evergreen Needleleaf Forest, Temperate Evergreen Needleleaf Woodland, Subtropical Evergreen Needleleaf Forest, Cool Needleleaf Forest |
| Temperate mixed forest | Temperate Cool Mixed Forest, Temperate Warm Mixed Forest, Temperate Cool Mixed Woodland, Temperate Warm Mixed Woodland |
| Subtropical mixed forest | Subtropical Mixed Forest, Subtropical Mixed Woodland |
| Other | Temperate Grassland, Subtropical Grassland, Temperate Shrubland, Subtropical Shrubland |
