# Supplementary figures and images for: Fire, CO2, and climate effects on modeled vegetation and carbon dynamics in western Oregon and Washington
Source: PLoS One. 2019 Jan 25;14(1):e0210989. doi: 10.1371/journal.pone.0210989 (PMC6347276; doi:10.1371/journal.pone.0210989)

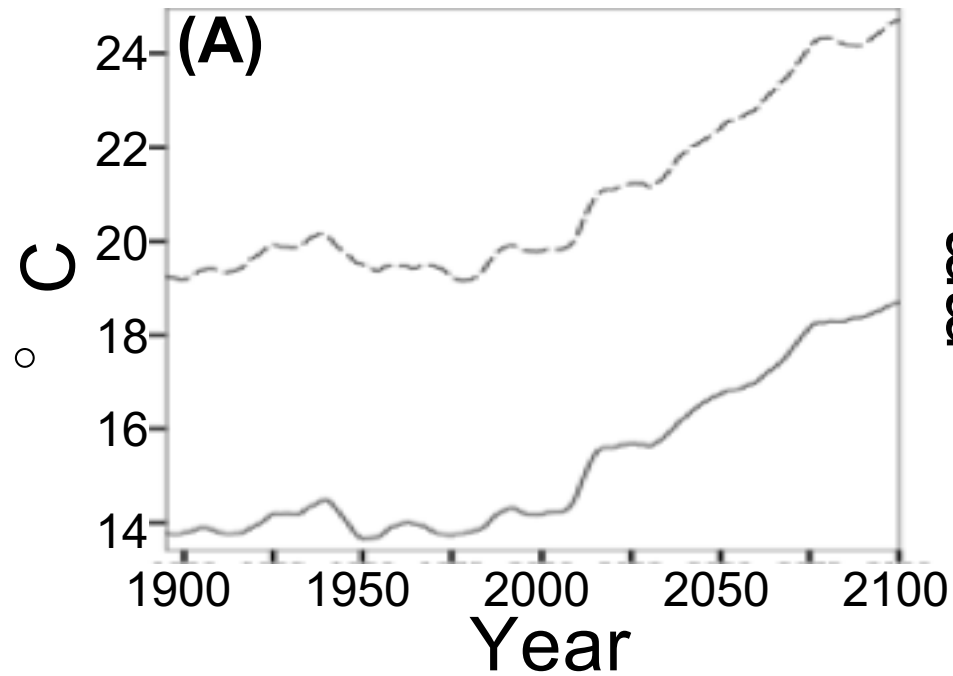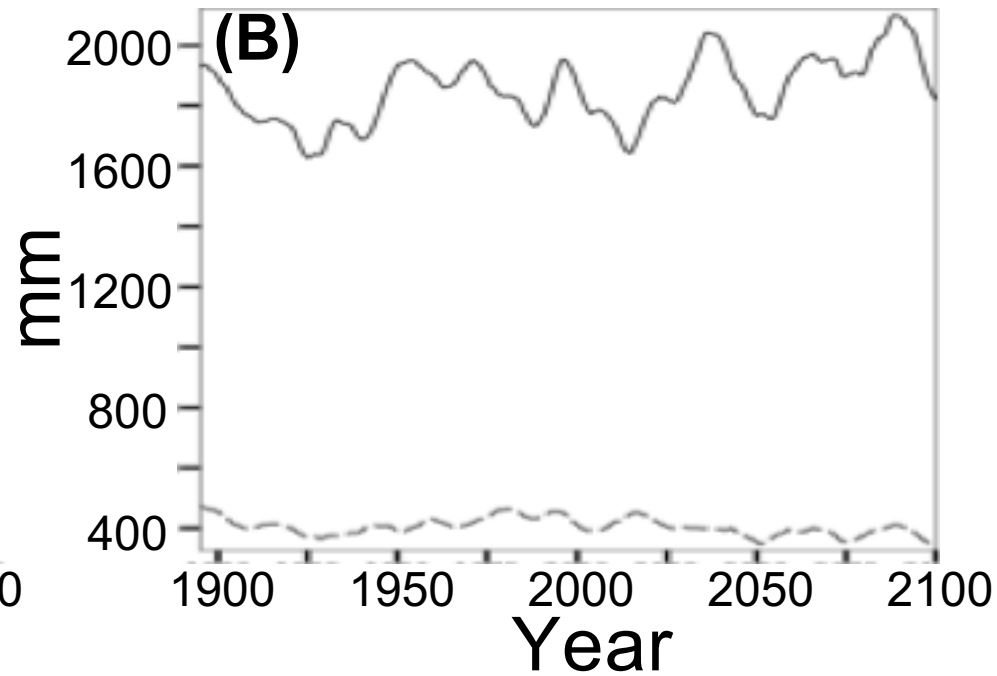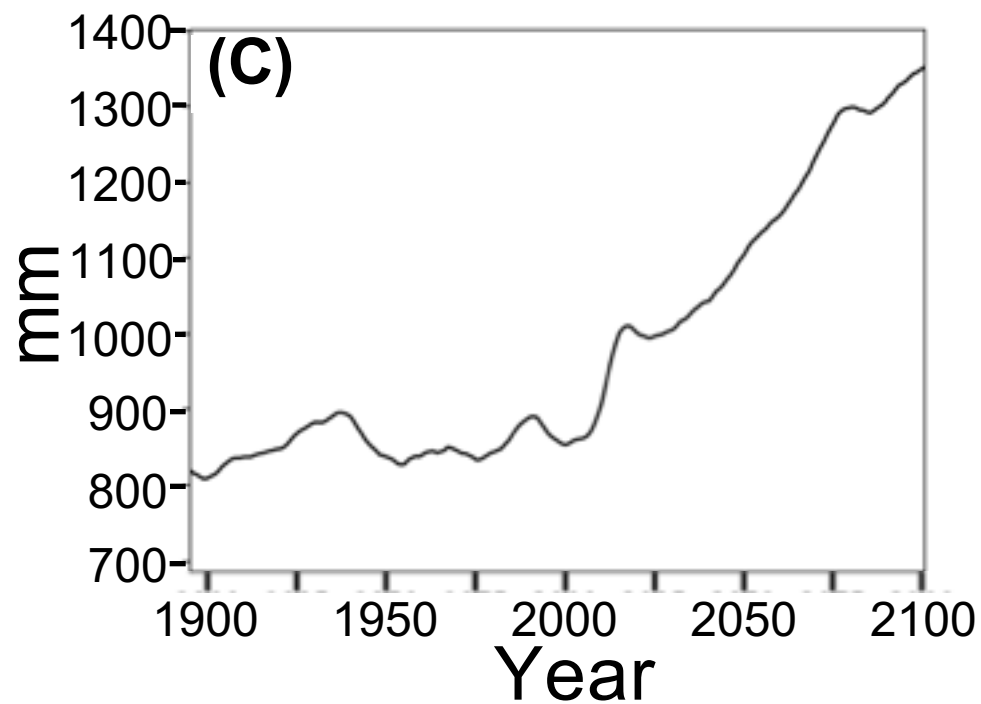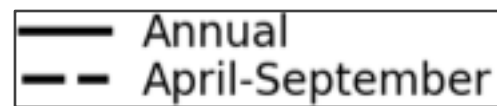

Supplement: S1 Fig — (A) CCSM4 RCP 8.5 annual and April-September maximum temperature, (B) CCSM4 RCP 8.5 annual and April-September precipitation, and (C) Annual PET calculated by MC2. All results smoothed using triangle smoothing +/- 8 years. (PDF) [file pone.0210989.s002.pdf]
