# Supplementary material for: Fire, CO2, and climate effects on modeled vegetation and carbon dynamics in western Oregon and Washington
Source: PLoS One. 2019 Jan 25;14(1):e0210989. doi: 10.1371/journal.pone.0210989 (PMC6347276; doi:10.1371/journal.pone.0210989)
Supplement: S2 Table — (DOCX) [file pone.0210989.s003.docx]

|  | **1895 - 2024** | **1936 - 1965** | **1971 - 2000** | **2036 - 2065** | **2071 - 2100** |
| --- | --- | --- | --- | --- | --- |
| **Annual Tmax (degC)** | 13.8  (0.45) | 14.0  (0.70) | 14.0  (0.54) | 16.7  (0.57) | 18.4  (0.52) |
| **April-Sep. Tmax (degC)** | 19.4  (0.68) | 19.6  (0.93) | 19.5  (0.66) | 22.4  (0.78) | 24.3  (0.59) |
| **Annual Tmin (degC)** | 3.1  (0.45) | 3.3  (0.56) | 3.5  (0.50) | 6.0  (0.56) | 7.5  (0.56) |
| **April-Sep. Tmin (degC)** | 6.2  (0.42) | 6.5  (0.50) | 6.8  (0.51) | 9.0  (0.62) | 10.7  (0.65) |
| **Annual Ppt (mm)** | 1795  (208) | 1838  (275) | 1874  (307) | 1906  (304) | 1978  (343) |
| **April-Sep. ppt(mm)** | 418  (83) | 410  (95) | 445  (86) | 378  (114) | 377  (84) |
| **Annual PET (cm)** | 832  (47) | 85  (72) | 855  (56) | 1107  (66) | 1305  (57) |

Temperature and precipitation are from PRISM and CCSM4 results used to drive the MC2 simulation, potential evapotranspiration was calculated by MC2. (Tmax: mean of monthly maximum temperature; Ppt: total precipitation; PET: potential evapotranspiration)
